# Current state of data stewardship tools in life science

**DOI:** 10.3389/fdata.2024.1428568

**Published:** 2024-09-16

**Authors:** Anna Aksenova, Anoop Johny, Tim Adams, Phil Gribbon, Marc Jacobs, Martin Hofmann-Apitius

**Affiliations:** ^1^Bonn-Aachen International Center for Information Technology (B-IT), University of Bonn, Bonn, Germany; ^2^Department of Bioinformatics, Fraunhofer Institute for Algorithms and Scientific Computing, Sankt Augustin, Germany; ^3^Fraunhofer Institute for Translational Medicine and Pharmacology, Discovery Research Screening Port, Hamburg, Germany

**Keywords:** data stewardship tools, FAIR, research data repositories, data sharing, literature review, data interoperability, mapping

## Abstract

In today's data-centric landscape, effective data stewardship is critical for facilitating scientific research and innovation. This article provides an overview of essential tools and frameworks for modern data stewardship practices. Over 300 tools were analyzed in this study, assessing their utility, relevance to data stewardship, and applicability within the life sciences domain.

## 1 Introduction

The term “data stewardship” is commonly used alongside “data governance” in the current literature (e.g., Brous et al., 2016; Rosenbaum, 2010), which can lead to confusion. Data stewardship covers the practical elements involved in managing and ensuring the quality of research data as assets, as well as ensuring that the data is accessible to the relevant community with high quality (Arend et al., 2022). Conversely, data governance refers to the establishment of policies, recommendations, concepts, and responsibilities for data stewardship (Rosenbaum, 2010).

Closely linked and directly related to the concept of “data stewardship” is the term FAIR (Wilkinson et al., 2016), which stands for Findability, Accessibility, Interoperability and Reusability. This term has been coined for a set of principles that are highly valid for scientific data. FAIR principles ensure research objects are reusable and accessible without specifying technical requirements. They promote rigorous evaluation and extensive reuse of data. FAIR guiding principles are not a standard but provide flexibility for different approaches to make data and services findable, accessible, and interoperable for reuse. Valuable standards can be developed, guided by the FAIR Principles.

Although FAIR data is a quite popular topic for discussion in the scientific community, our analysis for this review shows that the number of existing tools for FAIR data stewardship is remarkably small. Most FAIR projects are focused on “FAIRification” of existing repositories and promotion of FAIR principles, while the number of software solutions for FAIR data stewardship remains limited. An increased support infrastructure for FAIR data-publishing, analytics, computational capacity, virtual machines, and workflow systems is therefore necessary. Building infrastructures based on rich metadata that supports optimal reuse of research resources is a widely accepted goal, however, the implementation so far is not even coming close to achieving the ambitious goals of FAIR data in science (Mons et al., 2020).

Whilst FAIR principles have been thoroughly defined and worked out in sufficient detail (Mons et al., 2017), the concept of data stewardship is frequently misunderstood and confused with data governance and data management. There are concerns about the stretching of the original meanings of the FAIR Principles and confusion in their implementation (Jansen et al., 2019).

Despite the growing complexity of data, many researchers undertake FAIRification of their data themselves. However, they may lack expertise, knowledge, and experience in the field of data stewardship. In the domain of medicine, this is highly relevant: researchers are responsible not only for their findings, but for data stewardship including study design, data collection, analysis, storage, ensuring data quality and integrity, and sharing, as well as protecting the privacy of study subjects (typically patients). Whilst Research Institutes have a formal responsibility for sensitive data and are legally bound to appoint a Data Protection Officer to monitor GDPR compliance; there is no legal obligation to appoint data stewards (Jansen et al., 2019). Besides, the number of trained experts in the field of data stewardship is very limited, to say the least. This implies that researchers must assume the role of data stewards, despite potentially lacking expertise in this domain and having professional interests that are not typically aligned with data stewardship. Consequently, reusability of “patient-level data” is limited and significant effort needs to be invested to make medical study data FAIR in retrospective. To address this issue, specialized tools such as the ADataViewer for Alzheimer's disease have been developed (Salimi et al., 2022). ADataViewer establishes interoperability of longitudinal Alzheimer study data at variable level. However, such tools require an immense effort for data understanding, generation of a common data model (CDM) and mapping of individual variables to that CDM.

A lack of proper data stewardship demonstrable leads to data loss, lack of interoperability, lack of provenance and poor reuse of research data (Jansen et al., 2019). Many years of discussions on FAIR principles and their implementation through data stewardship have led to a change with funding bodies and research policy makers. Data stewardship is recognized as important for ensuring high-quality data and maximizing the “return on investment” by funding bodies (Wise et al., 2019). There is a clear consensus that data stewards should be involved as early as possible in publicly funded projects and should have expertise in all domains (Wise et al., 2019).

## 2 Data stewardship methods

**Figure 1:**
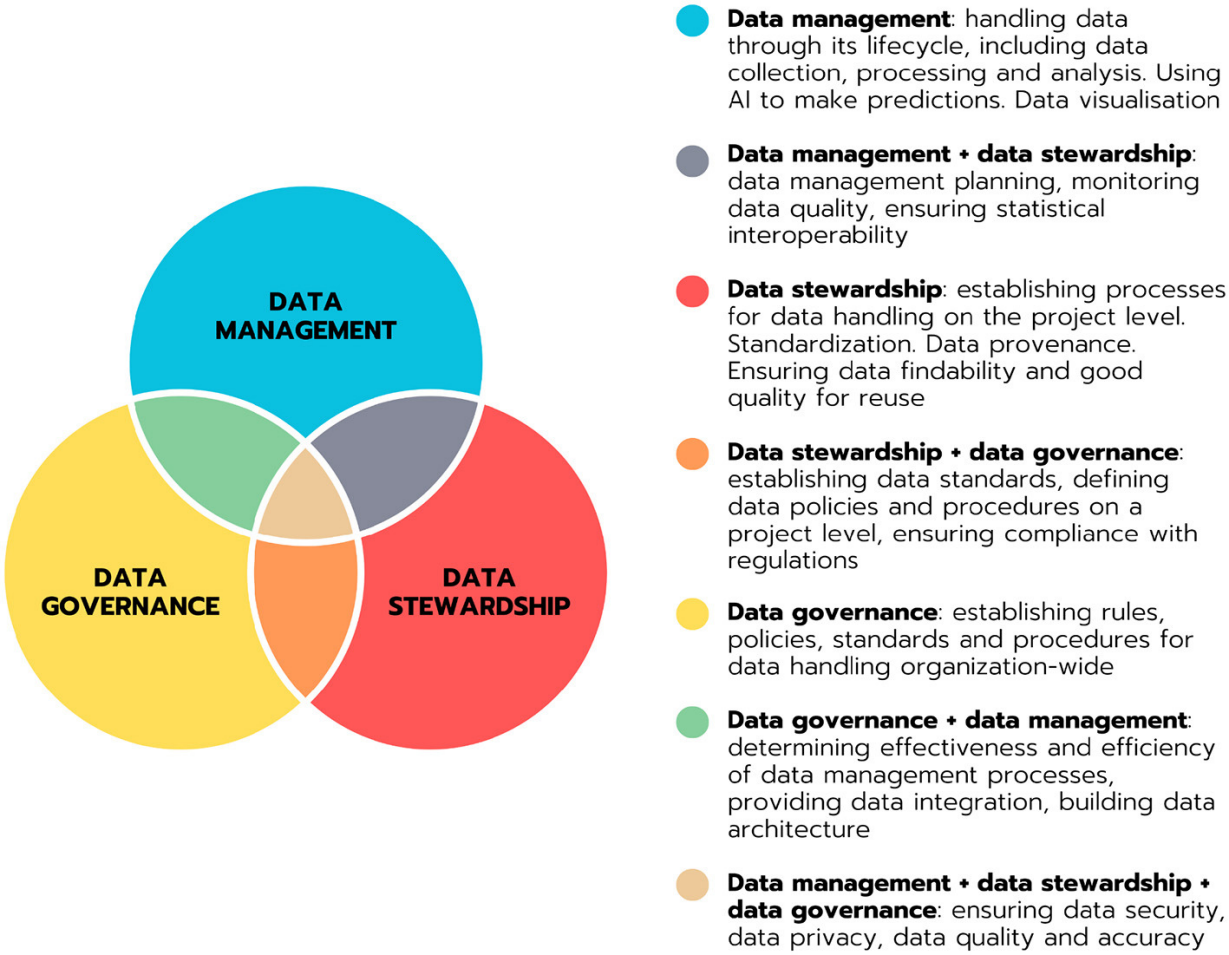
Venn diagram illustrating the domains of data management, data stewardship and data governance and their partial overlaps. Definitions of the domains and domain overlaps are provided.

Data stewardship plays a crucial role in the sustainable management of research data in clinical research. It encompasses methodologies that organizations use to ensure the responsible and ethical handling of data assets. However, the field of data stewardship is somehow fuzzy, broad and undefined, making it challenging to distinguish it from data management and data governance, which is illustrated in Figure 1.

**Figure 2:**
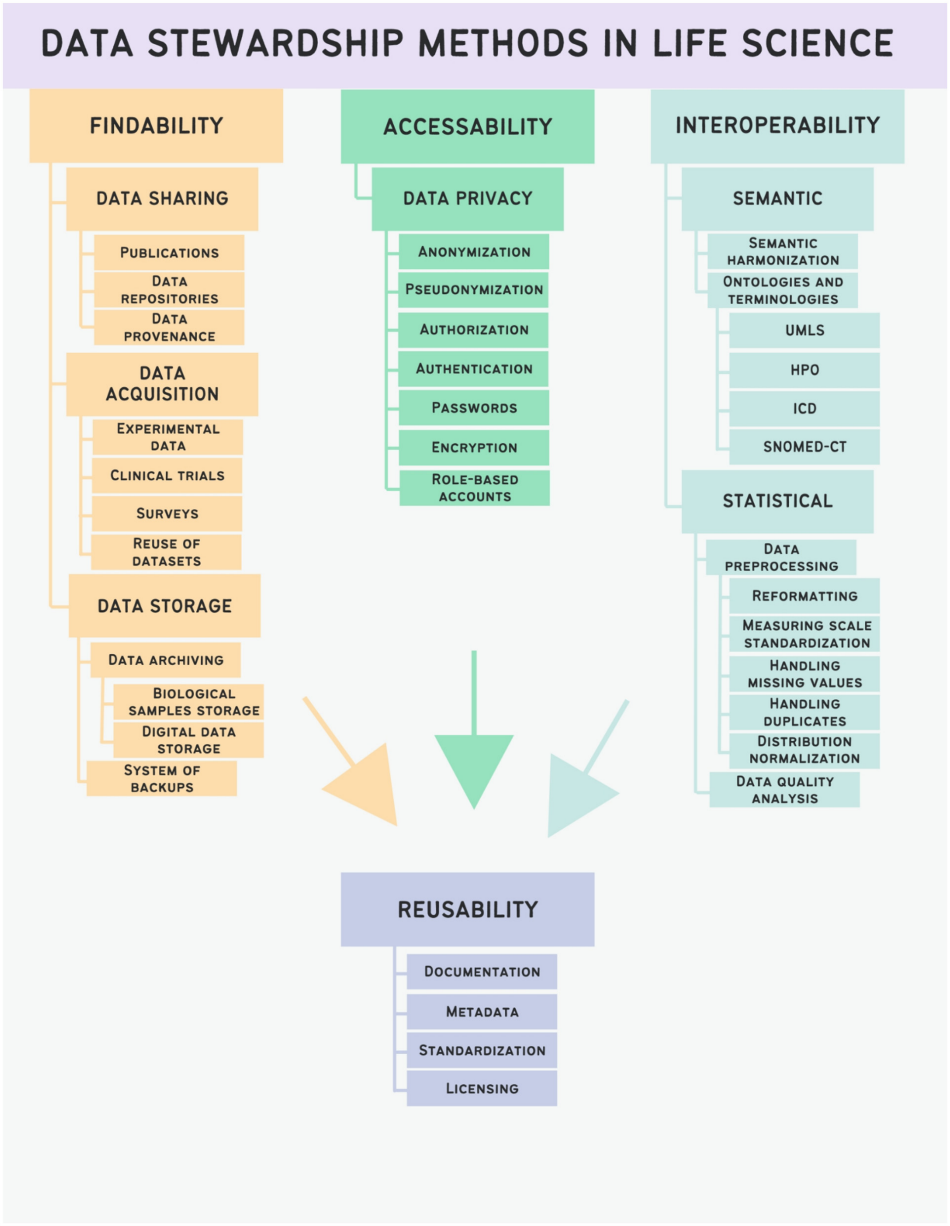
Schematic representation of data stewardship methods in life sciences.

This lack of clarity can lead to confusion regarding the specific practices that fall under data stewardship. In our research for this review, we tried to address this complexity by providing a clear schema in Figure 2.

Effective data stewardship methodologies are essential for maximizing the value and utility of data. Adequate data stewardship ensures that digital research data are FAIR in the long term. Data management, archiving, and reuse by third parties are all part of data stewardship, and it is a crucial part of Open Science. Adequate data stewardship protects the scientific integrity of research and meets requirements of research funders, journals, and makes sure that legal compliance to GDPR and other relevant laws is implemented.

In the following, we will discuss the individual data stewardship methods that—when combined—result in improved reusability of data and knowledge.

### 2.1 Findability

Important step of reusing data is to find it. It is crucial that both, humans and computers, can easily access data and the corresponding metadata. Machine-readable metadata plays a critical role in enabling automatic discovery of datasets and services, making this an indispensable part of the FAIRification process. Findability in data stewardship refers to the ability to easily locate and uniquely identify a specific data object or dataset. According to Wilkinson et al. (2016), this involves ensuring that data objects have unique and persistent identifiers, as well as machine-readable metadata that accurately describes the content and context of the data, and makes explicit the identifier of the data it describes. Both data and metadata must be registered or indexed as a searchable resource.

Data stewardship methods that ensure findability of research data include data cataloging, data sharing, structured description of methods for data acquisition and data identification processes.

#### 2.1.1 Data sharing

Sharing data fosters transparency in scientific research, enabling a comprehensive understanding of the analysis process and facilitating the reproducibility of results. Sharing data provides the basis for validation of machine learning models on independent data sets suitable for testing and validation. It thus provides the basis for generalization of insights gained through Machine Learning (ML) and Artificial Intelligence (AI) modeling. In the absence of comprehensive data, metadata, and details about the resources utilized to produce the data, reproducing a study becomes impossible (Uribe et al., 2022). Therefore, a lack of data sharing and data interoperability directly contributes to the reproducibility crisis we observe in biomedicine.

Data that is not shared within commercial organizations plays a crucial role in the development of intellectual property, ultimately resulting in economic gains. However, the publication of patents may serve to facilitate the long-term reuse of data. Still, challenges exist in terms of access to data, as well as the establishment of ontologies and standards within this domain.

Efficient sharing of data has the potential to amplify the advantages of costly and time-consuming large datasets (Wilson et al., 2021). Combining previously shared biological datasets accelerates the development of analytical techniques employed in biological data analysis. Furthermore, the reuse of rare samples enhances their impact. Aggregating data for meta-analyses increases the overall study power, while also reducing the occurrence of insulated, non-interoperable and unique (underpowered) studies. Moreover, data sharing enables researchers to build upon prior studies to confirm or challenge their findings, instead of duplicating the same experiments.

Data consists of recorded observations, while metadata describes the data itself and the methods used to generate it. In a life science context, metadata frequently includes supplementary details about biomedical samples (e.g., patient samples), such as sex, medical condition, and information about experimental equipment. Most biological disciplines adhere to specific metadata standards outlining the required information accompanying datasets.

Scientists seeking valuable guidance on appropriate data sharing practices are encouraged to refer to FAIRsharing. This collaborative platform consolidates information on standards, repositories, and policies aligned with the FAIR principles, providing domain-specific community standards (Sansone et al., 2019).

#### 2.1.2 Repositories

**Figure 3:**
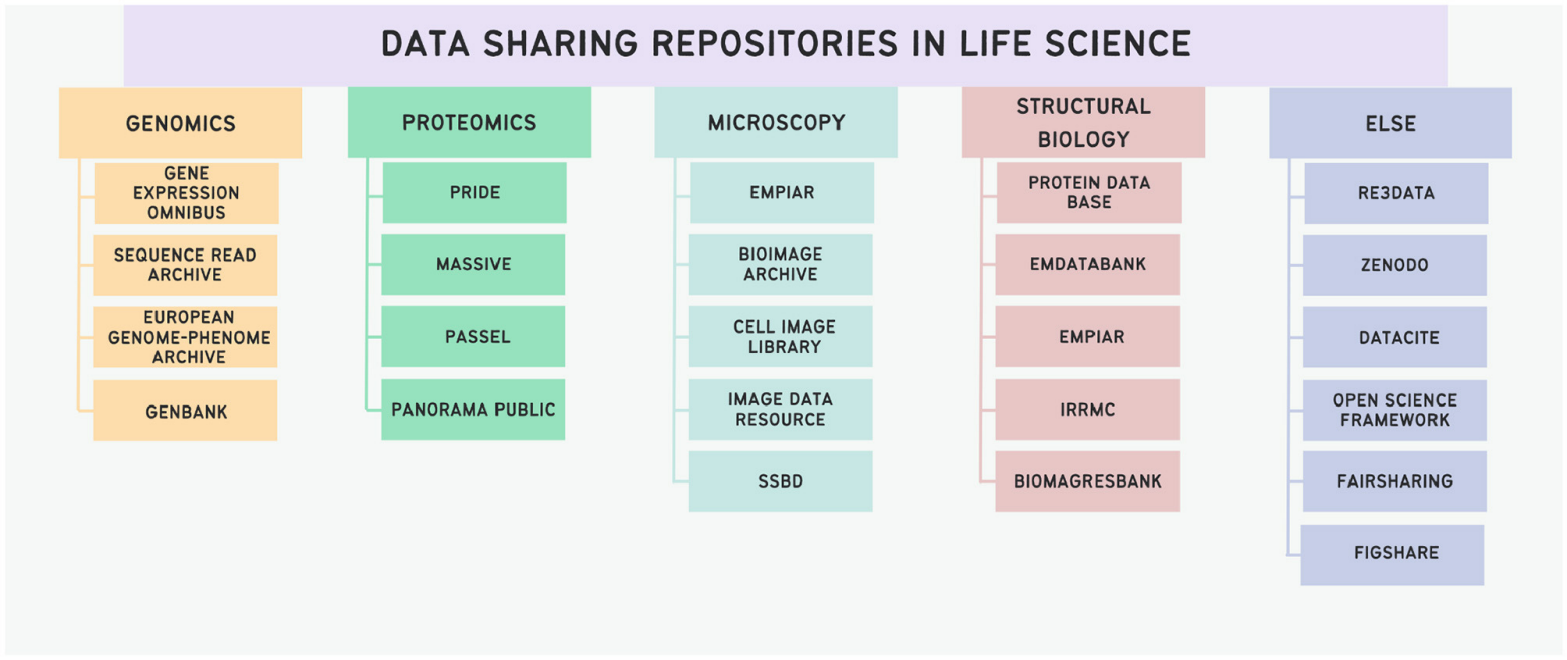
Schematic representation of data sharing repositories in life sciences. The ordering principle is based on the modality of data; with a dedicated class of general repositories.

There are numerous repositories where researchers can deposit their scientific data, and sometimes it can be challenging to find a repository suitable for a specific discipline. Repositories suitable for some areas of life science are presented in Figure 3.

Depending on the experimental nature, various specialized repositories cater to diverse data sharing needs, each imposing distinct requirements for data and metadata formatting. In cases where no repository aligns with the author's requirements, the generalist repository Zenodo can be employed for any type of scientific data (Sicilia et al., 2017). The Open Science Framework (OSF) also may serve as a generalist repository: this platform is used for structuring scientific projects. Additionally, OSF functions as a versatile repository, enabling the sharing of data and various materials by making the OSF project publicly accessible (Foster and Deardorff, 2017). To find appropriate repository to share the data, scientists may use FAIRsharing, the registry of standards, policies, knowledge bases and repositories (Sansone et al., 2019), or register of repositories re3data (Pampel et al., 2023). Other examples of general-purpose repositories are Dryad (Rousidis et al., 2014) and FigShare (Thelwall and Kousha, 2016).

The world of data repositories in the Life Sciences appears very heterogeneous and guiding principles concerning metadata annotations, legal guidance on data sharing or aspects like provenance cannot be found. First attempts at addressing these issues have been made (Wilson et al., 2021), but are not yet common practice.

#### 2.1.3 Identifiers

Identifiers serve the purpose of labeling, distinguishing, finding, and retrieving entities within a collection or resource managed by a specific organization, which acts as the authoritative body within the respective domain of knowledge. The fundamental idea is that identifiers must be distinct, meaning they must be assigned in a unique way. Consequently, there exists a one-to-one relationship between the identifier and the entity it represents. In isolated systems the likelihood of identifier collision is negligible. However, different isolated systems may create identical identifiers for different entities: these identifiers are considered locally unique, as there is no assurance of their uniqueness across all other existing systems, in other words, their global uniqueness cannot be guaranteed (Juty et al., 2020).

The FAIR principles advocate for the utilization of globally unique, persistent, and machine-resolvable identifiers (GUPRI or GUPRID) (Mangione et al., 2022) as essential components for both data and metadata. Every endeavor toward FAIRness is encouraged to employ tools and methodologies that facilitate the creation of unequivocal identifiers, ensuring their sustained functionality even in the event that the FAIRified assets are no longer accessible. Consequently, services and technologies for GUPRI should ensure the enduring availability of the identifiers associated with FAIRified assets.

Best citation practice requires globally unique and Internet-resolvable identifiers, specifically Uniform Resource Identifiers (URIs). URIs are concise character sequences uniquely identifying resources, which can be physical, digital, or abstract in nature. The scheme name in a URI indicates the resolution method, with a focus on secure http URIs. The resolution of URIs is explored within the context of REST interfaces, the standard method for accessing essential metadata, including resolution endpoints, for persistent identifiers.

Web-based identifiers have been in existence long before the emergence of FAIR principles. A very popular example is the Digital Object Identifier (DOI) system, for more than 20 years widely employed in publishing to identify documents and data sets, facilitating data citation and interoperability. Maintained by the International DOI Foundation, with DataCite as a crucial Registration Agency, over 16 million unique DOIs have been registered. DataCite membership allows organizations to mint DOIs annually, with resolution services provided at no cost (Juty et al., 2020).

While DOIs are valuable for uniquely identifying digital objects, pURLs (Persistent URLs) offer additional benefits in terms of granularity, customization, linking, and flexibility that make them valuable tools for managing and sharing digital resources effectively. PURLs can be easily managed and updated by organizations to reflect changes in the structure or content of digital resources. This flexibility allows for more dynamic linking and ensures that users are always directed to the most current version or location of a resource. DOIs are typically assigned at the level of a single digital object, such as an article or dataset. In contrast, pURLs can provide more granular identifiers that point to specific sections, components, or versions of a digital resource. This granularity can be useful for linking to specific parts of a resource or enabling more precise citations (Hakala, 2010).

Data stewards use digital identifiers to track and link data across different systems, ensuring data integrity and consistency. Identifiers are also used to enforce access controls, monitor data usage, and facilitate data sharing within and outside the organization. In order to uphold the principles of FAIR data management, it is imperative to assign digital identifiers to all published objects (Juty et al., 2020). These identifiers play a crucial role in enabling the tracking of data, ensuring its provenance, and enhancing its discoverability and reusability. By implementing digital identifiers, researchers can establish a robust framework for managing and sharing data effectively within the scientific community.

### 2.2 Accessibility

According to FAIR principles, data is considered accessible if it can be obtained by machines and humans upon appropriate authorization and through a well-defined, explicit protocol. Achieving optimal accessibility involves the utilization of linked metadata that describes datasets within central data repositories. By employing such linked metadata, researchers gain access to associated datasets, enhancing the effectiveness of their search results. These linked metadata conform to a standardized set of descriptions specific to biology. Public repositories typically offer the necessary technical infrastructure, access interfaces, and comprehensive documentation to facilitate proper usage (Fillinger et al., 2019). Making data available allows for verification of results, facilitates further analysis, and enables the reuse of data for new discoveries, ultimately advancing scientific knowledge and accelerating research progress (Veitch et al., 2022).

Ensuring data accessibility is closely connected with data privacy that restricts access to certain data for ethical reasons. This includes data anonymization or pseudonymization, passwords and encryption, authentication and authorization, role-based accounts for data access and following data privacy regulations that are different in healthcare and research, informed consent issues, intellectual property issues and the General Data Protection Regulation (GDPR).

Differential privacy is a framework for ensuring that the inclusion or exclusion of an individual's data in a dataset does not significantly affect the outcome of any analysis or query performed on that dataset. It aims to provide strong privacy guarantees by adding noise or randomness to query results, thereby obscuring the contribution of individual data points while still allowing accurate aggregate analysis (Ziller et al., 2021).

The GDPR is a comprehensive set of regulations that have been introduced to safeguard the personal data of European Union citizens. It establishes strict rules for the processing of personal data. One of the key aspects of the GDPR is the recognition of a special category of personal data known as health data (Lopes et al., 2020). Health data refers to any information related to an individual's physical or mental health, including medical history, diagnoses, treatments, and test results. This type of data is considered sensitive and is subject to special conditions regarding its treatment and access by third parties. This means that health data is subject to even stricter regulations than other types of personal data. The GDPR requires organizations that work with health data to obtain explicit consent from individuals before doing so. Additionally, organizations must implement appropriate measures to ensure the confidentiality of health data. They must also appoint a Data Protection Officer (DPO) to oversee compliance with GDPR regulations.

Besides compliance with GDPR regulations, compliance with ISO 27001 standards is strongly recommended for any type of sensitive data. ISO 27001 is a standard that helps organizations establish a secure and reliable method of network communication (Wylde et al., 2022). This includes implementing protocols for data access control and encryption of passwords to prevent unauthorized access to sensitive information. The standard also emphasizes the importance of training cybersecurity staff to be able to detect and respond to potential attacks from malicious third parties. By adhering to ISO 27001, organizations can minimize the risk of network communication attacks and ensure that their data remains safe and secure.

It is important to note that protected data that is not available for common free use is still considered accessible. There are valid reasons for keeping data shielded from public access, and one of them is competitiveness. The FAIR principles emphasize the importance of providing transparent details on how to access data, the context in which the data was generated, guidelines for reuse, and proper citation instructions. However, FAIR does not enforce data to be openly accessible or free of charge. Data that is not available for free is still considered accessible because there is a publicly available protocol that enables user to access data through payment. Accessible data is not equal to open data and does not guarantee that data will be available for free and for every user (Mons et al., 2017).

### 2.3 Interoperability

According to FAIR data principles, interoperability refers to the integration and collaboration of data or tools from disparate sources, requiring minimal effort. To be interoperable, data and metadata should use formal language broadly applicable for knowledge representation and should use vocabularies that follow FAIR principles. Also data and metadata should include qualified references to other data (Wilkinson et al., 2016). It is noteworthy that even though those requirements provide semantic data interoperability, they do not establish any statistical data interoperability.

FAIR principles prioritize machine-actionability to fully adhere to FAIR guidelines. Although RDF and ontologies are commonly used to meet FAIR criteria, other data formats tailored to specific needs can also be applied in a FAIR framework. Utilizing RDF with appropriate ontologies is called semantic harmonization. It enhances interoperability and facilitates information exchange, especially at the metadata level (Mons et al., 2017).

Achieving data interoperability at semantic level can be realized through various methods, including the creation and adherence to controlled vocabularies (CVs), standardized chemical nomenclature, and compliance with formatting standards for the exchange of data (Vesteghem et al., 2020). The goal of semantic harmonization is to provide a common vocabulary for research, where each term has a clear and unambiguous meaning. Usage of common terminology allows smooth integration and machine readability. There are several approaches to this task, such as using shared standards, terminologies, and ontologies. The Ontology Lookup Service offered by EMBL-EBI offers a user-friendly search platform for ontologies (Vesteghem et al., 2020).

SNOMED CT is widely recognized as a standardized system for naming healthcare concepts globally. It is one of the largest and most robust ontologies, which serves as a coding system for term identification and a multi-hierarchical ontology that facilitates the relationship between concepts. Managed by the International Health Terminology Standards Development Organization (IHTSDO), now known as SNOMED International, SNOMED CT is a comprehensive clinical terminology system that offers a standardized approach to representing clinical data collected by healthcare workers (Chang and Mostafa, 2021).

Alternative approach is the use of UMLS. The UMLS Metathesaurus, the most extensive thesaurus in the biomedical field, offers a structured representation of biomedical information, organizing concepts based on semantic type and establishing both hierarchical and non-hierarchical connections between them (Aronson, 2001).

There are many existing ontologies for different fields of research that can be found using Ontology Lookup Service. One of the most popular ontologies in the field of bioinformatics in Human Phenotype Ontology (HPO). HPO methodically defines and categorizes human phenotypes in a logical manner. Serving as an ontology, HPO facilitates computational reasoning and advanced algorithms to aid in integrated genomic and phenotypic analyses. The extensive clinical, translational, and research uses of HPO encompass genomic interpretation for diagnostic purposes, gene-disease identification, mechanism elucidation, and cohort analysis, all contributing to the advancement of precision medicine (Köhler et al., 2021).

Another well-known source of common terms is the International Classification of Diseases (ICD). For more than a century, the ICD has served as the primary foundation for ensuring the comparability of statistics related to mortality and morbidity causes across different locations and throughout various time periods. A significant amount of knowledge regarding the prevalence, origins, and impacts of human illnesses globally relies on data organized according to the ICD. Clinical adaptations of the ICD form the primary framework for disease statistics, especially pertaining to hospital-treated cases. These statistics play a vital role in essential functions like payment structures, service strategizing, quality control and safety management, as well as health services research (Harrison et al., 2021).

Implementing ontologies, classifications and terminologies at early stages of the data collection process improves interoperability and findability (Vesteghem et al., 2020). The use of common vocabularies enables ontology mappings and content mappings. It is noteworthy to mention that mapping as a data stewardship challenge may face a revolution through the utility of Large Language Models and their embeddings (Salimi et al., 2024).

### 2.4 Reusability

In the context of FAIR data principles, reusability is a fundamental aspect that emphasizes making data easily understandable and accessible for future use for different purposes beyond its original intent. This can include applications in new research projects, policy-making, education, or commercial use. To enhance reusability, data should be well-documented with clear descriptions of the methodology, context, and conditions for reuse. This ensures that others can confidently interpret and apply the data in different contexts (Wilkinson et al., 2016). Additionally, providing appropriate metadata, standardized formats, and clear licensing information contributes to the reusability of data.

Metadata should describe the data's context, methodology, quality, and any transformations applied to it. This allows users to understand how the data was collected and processed.

High-quality data that has been validated and curated is more likely to be reused. Ensuring accuracy and reliability enhances trust in the data.

By adhering to FAIR principles, researchers and organizations can foster a culture of data sharing and maximize the potential for meaningful and impactful reuse of data across various disciplines.

All the above-mentioned principles (findability, accessibility, and interoperability) serve to provide better reusability of scientific data. There are no specific data stewardship methods that directly address reusability: it is the combination of findability, interoperability and accessibility methods that results in offering better reusability.

## 3 Data stewardship tools and services

Given the expansive nature of data stewardship and its nebulous boundaries, distinguishing between tools that qualify as data stewardship tools and those that do not does pose a challenge. Certain researchers view data stewardship as a broad concept encompassing a wide array of data management tools. In this paper, we have curated a selection of tools based on the following criteria:

1. The tool under consideration must be designed for applications within the life sciences domain or demonstrate utilization within this field (documented by publications).
2. Adherence to FAIR principles in the design of the tool or incorporation of key features conducive to FAIR data stewardship is essential for inclusion.
3. The tool's availability as of March 2024 is a prerequisite for its consideration in this study.

**Figure 4:**
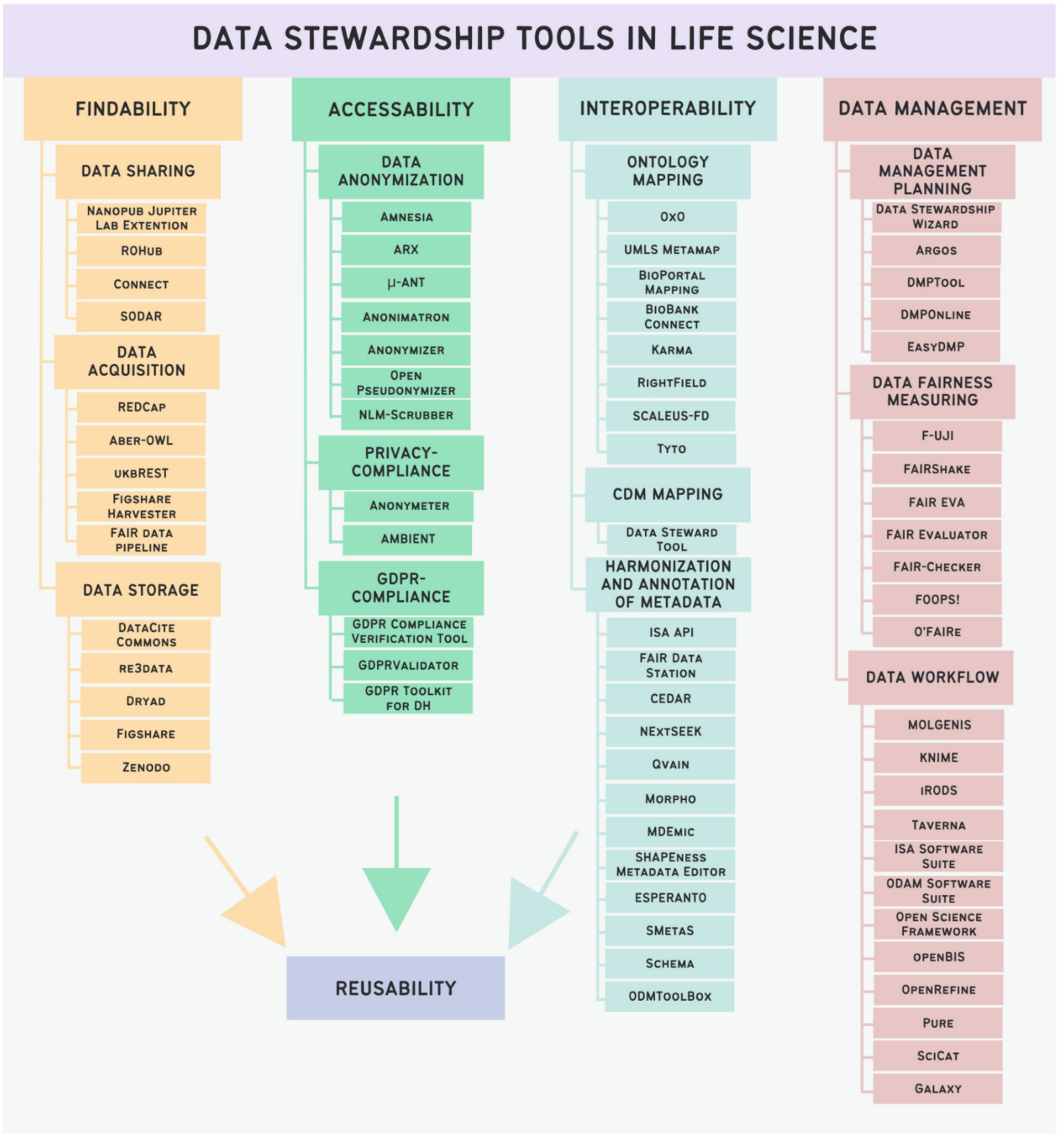
Categorization of data stewardship tools in Life Science and listing of instances of selected tools.

A total of over 300 tools were initially gathered from publications related to the field and retrieved through searches on PubMed. Many tools were gained from the publication of Mangione et al. (2022), where 277 tools were analyzed, but most tools were not related directly to life sciences. Following the application of rigorous selection criteria mentioned above, 70 tools were identified and are listed in Figure 4. The tools have been categorized based on their intended function into Findability tools, Accessibility tools, and Interoperability tools. While data management and data stewardship are distinct topics, a subset of data management tools has been incorporated due to the integral role of effective data management in facilitating robust data stewardship practices. Additionally, certain tools tailored for data stewardship purposes may align with the domain of data management, underscoring the interconnected nature of these disciplines.

### 3.1 Findability tools

We categorized tools facilitating Findability into three primary groups: data sharing tools, data acquisition tools, and data storage tools.

**Data sharing tools** streamline the process for scientists to share their data in alignment with FAIR principles, as the name suggests. These tools are essential for promoting collaboration, transparency, and reproducibility in scientific research.

The Nanopub Jupiter Lab Extension, a tool developed by the FAIR Workflows project, represents a significant advancement in the field of scientific data management. This Jupiter Lab extension is specifically tailored to facilitate the searching and publishing of Nanopublications within the Python notebook environment (Mangione et al., 2022).

ROHub serves as a research object management platform aimed at facilitating the preservation and lifecycle management of scientific investigations. Notably, it adopts the research object model and paradigm as its core framework. This involves consolidating resources linked to a specific experiment into a singular digital entity referred to as a “research object.” Additionally, essential metadata is presented in a semantic format, accessible to both users and machines, providing a comprehensive approach to organizing and interpreting scientific content. ROHub contributes to supporting scientists and researchers in the effective oversight and safeguarding of their research efforts, as well as facilitating the sharing and publication of their work (Palma et al., 2014).

The CONNECT dashboard, developed by OpenAIRE, simplifies the process of publication and sharing the research data, applying Open Science principles (Príncipe et al., 2017).

The SODAR tool offers a versatile solution for scientists, enabling seamless metadata management through the ISA API, efficient data storage via iRODS, and comprehensive data acquisition and analysis functionalities. By leveraging SODAR, researchers can effectively curate their data for publication and dissemination in alignment with the FAIR principles, thereby enhancing the accessibility and reusability of their scientific findings (Nieminen et al., 2022).

**Data acquisition tools** are used to find related datasets and publications, check data provenance and gather additional information. These tools are essential for gathering accurate and reliable data in a fast and convenient way.

REDCap introduces an innovative workflow methodology and software solution created to expedite the development and implementation of electronic data capture tools, specifically tailored to bolster clinical and translational research efforts. It is a web-based application that serves as a valuable facilitator in the development of clinical research, predominantly within health-related domains, thereby contributing to a reduction in research costs. It empowers researchers to optimize usage of electronic data capture components. The adoption of REDCap enhances the methodologies employed in data collection while concurrently offering a secure repository for data storage. Established as a robust instrument for clinical research, REDCap has garnered widespread utilization by academic and governmental institutions (Harris et al., 2009; Garcia and Abrahão, 2021).

The Aber-OWL infrastructure furnishes reasoning services tailored for bio-ontologies. Comprising an ontology repository alongside a suite of web services and interfaces, Aber-OWL facilitates ontology-driven semantic access to and retrieval of biological data and literature within the domain (Hoehndorf et al., 2015).

The ukbREST tool has been specifically developed for the UK Biobank study, with potential for adaptation to other biobanks, providing users with streamlined access to phenotypic and genetic data. Through its REST API, ukbREST enables efficient retrieval of data within a secure network environment. These features position ukbREST as a useful resource in enhancing the accessibility of valuable biobank data to the scientific community, thereby fostering reproducibility in research analyses (Pividori and Im, 2019).

The Figshare Harvester for OpenVIVO constitutes a specialized tool crafted to aggregate data from Figshare. This harvester is equipped to conduct data harvesting based on tags or ORCiD identifiers. When provided with a designated tag or identifier, the harvester systematically compiles all content associated with that query from Figshare, generating RDF for each individual work. Notably, the Harvester adheres to openVIVO URI conventions for dates and individuals, ensuring that only distinctly identified works and individuals are incorporated into the resulting RDF dataset (Ilik et al., 2018).

The FAIR Data Pipeline (Mitchell et al., 2022) was developed amidst the COVID-19 crisis to offer a valuable resource for tracking provenance within the scientific community. This tool effectively synchronizes data and metadata between the execution platform and a remote data registry, simplifying the process of tracing the origins and history of scientific data and gathering related information.

**Data storage tools** are designed for secure long-term preservation of datasets, metadata and related information. Effective data storage solutions not only safeguard the data against loss or corruption but also facilitate easy retrieval and sharing among the scientific community. Due to the high number of different scientific repositories for various domains in life sciences, only the most popular universal repositories are mentioned in this section.

DataCite (Neumann and Brase, 2014) was formed to address the complexities associated with data citation. Its primary objectives include facilitating seamless data access, promoting the adoption of data publication practices, and endorsing data archiving efforts. Usage of DOIs offers a straightforward mechanism for retrieving and reusing research data.

Re3data (Pampel et al., 2023) is a global registry of research data repositories, used by scientists to find an appropriate repository to store and share research data. Presently, re3data delineates the characteristics of more than 3,000 research data repositories utilizing a comprehensive metadata schema and provides numerous opportunities for searching.

Dryad is an open-access repository that allows scientists to securely store, search, access, and reuse research data associated with their scholarly publications (Rousidis et al., 2014). By submitting data files with DOIs and metadata, researchers can streamline the procss of data discovery and preservation. The primary goal of Dryad is to facilitate the accessibility and reusability of valuable research data, ultimately enhancing the impact of scientific findings.

Figshare (see https://figshare.com) differs from other repositories as it allows researchers to share datasets that were not published. FigShare accommodates the uploading of diverse data categories by contributors, encompassing data that is in the pre-peer review phase. This facilitates immediate distribution, giving authors the opportunity to get early feedback. A growing number of esteemed journals have embraced the publication of preprints to streamline the peer review process. Furthermore, FigShare actively promotes the dissemination of negative results and research data that has been generated but remains unreported, fostering a more comprehensive and transparent scientific discourse (Thelwall and Kousha, 2016).

Zenodo (see https://zenodo.org) is a research data repository and digital preservation platform that provides a reliable and open-access space for researchers to store and share their research outputs. Developed by OpenAIRE and CERN, Zenodo accommodates a wide range of research data, including datasets, software, publications, and project documentation. As an integral part of the European Open Science Cloud (EOSC), Zenodo contributes to the global open science movement by facilitating the curation and accessibility of research data (Sicilia et al., 2017).

### 3.2 Accessibility tools

Within the realm of accessibility tools, two primary categories have been identified: anonymization and pseudonymization tools, privacy-compliance verification tools and GDPR-compliance verification tools. **Anonymization and pseudonymization tools** play a crucial role in safeguarding sensitive patient data for subsequent publication.

Amnesia, developed by OpenAIRE, serves as a data anonymization tool designed to facilitate the anonymization of sensitive data, thereby enabling subsequent statistical analysis. This tool is used to avoid the risk of deanonymization while concurrently minimizing any potential degradation in data quality (Crutzen et al., 2019).

μ-ANT represents a pragmatic and readily configurable anonymization tool tailored for healthcare data. Through the integration of contemporary methodologies, it ensures robust privacy assurances while endeavoring to maintain the utility of anonymized data. Notably, μ-ANT accommodates the diverse attribute types prevalent in electronic healthcare records, catering to the needs of both practitioners and software developers engaged in data anonymization efforts (Sánchez et al., 2020).

Anonimatron is a free open-source data anonymization tool. It recognizes patterns in the dataset and generates anonymized datasets for further use (Kulkarni and Bedekar, 2022).

Anonymizer stands as another open-source tool designed for data anonymization. Employing anonymized random data, it systematically replaces all information within a database. A distinctive attribute of Anonymizer lies in its emphasis on data formatting, ensuring that the generated data closely mirrors the structure of the original data from authentic users (Vovk et al., 2023).

NLM-Scrubber, an openly accessible clinical text de-identification tool, has been developed by the National Library of Medicine. Its objective is to empower clinical scientists with access to health information devoid of patient identification, adhering to the Safe Harbor principles articulated in the HIPAA Privacy Rule (Kayaalp et al., 2015).

**Privacy-compliance verification tools** are used to measure the risks of privacy breaches or deanonymization.

Anonymeter is a statistical framework for quantifying privacy risks in synthetic tabular datasets, focusing on singling out, linkability, and inference risks (Giomi et al., 2022). Through experiments, they show that privacy risks scale with the amount of privacy leakage, with synthetic data exhibiting low vulnerability to linkability. Anonymeter outperforms existing frameworks in detecting privacy leaks and computation speed, contributing to a privacy-conscious use of synthetic data.

The Automated Cyber and Privacy Risk Management Toolkit (AMBIENT) assesses and analyzes an organization's cyber and privacy risks, providing recommendations for mitigating measures that optimize risk reduction (Gonzalez-Granadillo et al., 2021). Comprising three primary modules, AMBIENT includes a Cybersecurity Risk Assessment module for evaluating potential cyber threat scenarios, a Privacy Risk Assessment module for identifying privacy risks in compliance with GDPR objectives, and a Risk Mitigation module for selecting and implementing optimal measures to address identified risks.

**GDPR compliance verification tools** are instrumental in ensuring adherence to GDPR guidelines. These tools assess and monitor organizational practices to verify the proper handling and protection of personal data in accordance with GDPR requirements.

GDPRValidator endeavors to support small and medium-sized enterprises that have transitioned their services, wholly or partially, to cloud environments in achieving compliance with GDPR. This tool specifically addresses the challenges encountered in managing and storing data within cloud infrastructures, ensuring alignment with GDPR standards (Cambronero et al., 2022).

The Automated GDPR Compliance Verification Tool represents a scalable data protection solution designed with a focus on automated compliance verification and auditability, rooted in informed consent and modeled through a knowledge graph. This tool achieves automated compliance verification by employing a regulation-to-code process, translating GDPR regulations into precisely defined technical and organizational measures, culminating in the generation of software code. This approach ensures a systematic and efficient integration of GDPR principles into the design and implementation of data protection measures (Chhetri et al., 2022).

Ensuring compliance with GDPR holds particular significance within the digital health domain, given the commonplace processing of highly sensitive personal health data. To streamline the intricate process of compliance with regulations, the GDPR Toolkit for Digital Health has been developed with the explicit intention of providing support and guidance in navigating the complexities associated with GDPR adherence in the realm of digital health (Hussein et al., 2022).

### 3.3 Interoperability tools

In the spectrum of interoperability tools, we have categorized three principal types: ontology mapping tools that enhance interoperability by aligning datasets with established ontologies, tools for mapping datasets to common data models, and tools dedicated to the harmonization and annotation of metadata.

**Ontology mapping tools** are commonly used to identify terms within ontologies that correspond to data and metadata. This process creates a unified set of variables across different datasets. This mechanism ensures enhanced interoperability by fostering a common understanding of variables and facilitating seamless integration of diverse datasets.

The OxO tool, developed by EMBL-EBI, functions as a service dedicated to identifying mappings between terms derived from ontologies, vocabularies, and coding standards. It facilitates this process by importing mappings from diverse sources, including the Ontology Lookup Service and a selected subset of mappings from the UMLS (Harrow et al., 2020).

UMLS MetaMap, developed by the National Library of Medicine, is a program designed to map biomedical text onto the Metathesaurus or, equivalently, to discern concepts within the Metathesaurus referenced in the text. Employing a knowledge-intensive approach, MetaMap leverages symbolic, natural language processing, and computational linguistic techniques to achieve accurate and contextually informed mappings (Aronson, 2001).

The BioPortal Mapping terminology service offers a unified interface to access diverse terminologies and ontologies. Leveraging BioPortal's scalable infrastructure, this service enhances performance while concurrently minimizing maintenance costs, providing an efficient and feature-rich platform for navigating and utilizing various terminological resources (Zhao et al., 2016).

BiobankConnect (Pang et al., 2015) offers a user-friendly interface designed to expedite the biobank harmonization process, presenting a streamlined approach. Its applicability extends beyond biobank operations, making it a potentially valuable tool for various biomedical data integration endeavors.

Karma (Erkimbaev et al., 2018) serves as an information integration tool, facilitating swift and seamless integration of data from diverse sources such as databases, spreadsheets, and text files. Utilizing a graphical user interface, users can model information based on a selected ontology, automating a substantial portion of the integration process. The tool employs machine learning to discern the mapping of data to ontology classes, subsequently proposing a model that effectively links these classes together.

RightField, an open-source tool (Wolstencroft et al., 2011), facilitates the integration of ontology term selection into Excel spreadsheets. Although developed prior to the FAIR principles, RightField remains a valuable asset in the realm of data stewardship. Its primary function involves the creation of semantically aware Excel spreadsheet templates, which scientists can then reuse for data collection and annotation. Notably, scientists benefit from RightField by consistently annotating their data without delving into the intricacies of various standards and ontologies, all seamlessly embedded within the familiar Excel spreadsheet environment. This approach ensures data consistency without necessitating a departure from customary scientific practices.

SCALEUS-FD (Pereira et al., 2020) is a semantic web tool that complies with FAIR Data principles, allowing for data integration and reuse through online exposure of data and metadata in a self-descriptive manner. The tool addresses privacy issues and enables cataloging and searchability, with potential for improving medical care, public health policies, and clinical trials. Semantic technologies are able to describe data, map and link distributed datasets, and create an information network that can be used to search for information from a single entrypoint. FAIR data requires a persistent, globally unique identifier for data and metadata, as well as rich and standardized metadata that includes clear references to the identified data. The FAIRification process involves transforming data into a machine-readable, FAIR-compliant representation, defining metadata on data usage and provenance, and providing a query interface for end-users. SCALEUS allows migration of structured and unstructured information into a semantic format without the need for a predefined data integration ontology, offering flexibility in managing data models.

Take Your Terms from Ontologies (Tyto) (Bartley, 2022) is a lightweight Python tool designed to facilitate the incorporation of controlled vocabularies into routine scripting practices. Initially developed for applications in synthetic biology, Tyto showcases versatility that may prove beneficial for users engaged in diverse areas of bioinformatics research.

The exclusive utilization of the **CDM mapping** approach is observed in a singular tool, namely the Data Steward Tool (DST). CDMs are standardized structures or formats used to organize and represent data from various sources in a consistent manner. CDMs provide a common framework for describing data elements, relationships, and attributes, regardless of the original source or format of the data, enabling cataloging, managing data, and improving interoperability across resources. Domain-specific common data models benefit research institutions and facilitate data sharing.

It is noteworthy at this point to mention that the availability of CDMs and the mapping capability to CDMs is a prerequisite for federated learning (Oh and Nadkarni, 2023).

The CDM mapping is the process of aligning data from various sources to a standardized model. In this approach, data elements, attributes, and relationships from different datasets are mapped to corresponding elements in the CDM. CDM mapping typically involves identifying similarities and differences between the structure and semantics of data in different datasets and mapping them to the corresponding elements in the CDM. This process may require data transformation, normalization, or standardization to ensure compatibility and consistency across datasets.

DST is an application that allows for semi-automatic semantic integration of clinical data into ontologies and global data models and data standards. DST can standardize clinical datasets, map them to ontologies, and align with OMOP standards. DST is a web application for clinical data management and visualization. It provides a user-friendly interface to extend the model, add mappings, and read clinical data (Wegner et al., 2022). The COVID-19 pandemic has generated a vast amount of heterogeneous clinical data worldwide. Establishing a CDM specific to COVID-19 and using tools like DST can facilitate standardization and normalization of these datasets. By unifying and standardizing the data, data scientists can analyze larger cohorts. The COVID-19 CDM, developed in the COPERIMO Plus project, incorporates multiple datasets and can export standardized data to FHIR format. The DST is used for mapping data from various sources, enriching the CDM, and comparing with other global data standards like OMOP (Wegner et al., 2022).

**Tools for metadata harmonization and annotation** play a crucial role in establishing interoperability through the alignment of metadata. These tools enable editing of metadata, ensuring a harmonized and standardized approach to enhance compatibility across diverse datasets.

One prominent tool within this category is the ISA API, an integral component of the ISA Software Suite. The ISA API offers users robust programmatic capabilities for handling metadata, facilitating automation through a standardized interface. It acts as a key interoperable link between the two ISA formats and integrates seamlessly with various life science data formats essential for depositing data in public databases (Johnson et al., 2021).

The FAIR Data Station (Nijsse et al., 2022) offers tools for the proper FAIRification of (omics) data and provides capabilities to construct searchable metadata databases for similar projects. Furthermore, it offers assistance in the submission of sequence data metadata to the European Nucleotide Archive (ENA).

CEDAR, an acronym for the Center for Expanded Data Annotation and Retrieval (Vesteghem et al., 2020), offers a comprehensive suite of freely available tools. These tools encompass the creation of metadata templates, the population of templates with metadata, the submission of metadata to external repositories, and the storage, search, and management of both templates and metadata (Musen et al., 2015).

Qvain (Keskitalo and van Hemel, 2018), developed as part of the Finnish project Fairdata, is a tool designed to streamline the creation of standardized metadata for research datasets. As an integral component of the Fairdata services, Qvain offers workflows to facilitate the generation of structured metadata, enhancing the overall quality of research datasets. Qvain is an open-source project (see https://www.fairdata.fi/en/about-fairdata/fairdata-services/).

Morpho, a desktop application (Higgins et al., 2002), empowers researchers in the field of ecology and earth sciences to generate metadata and construct a catalog encompassing both data and metadata. Developed by The Knowledge Network for Biocomplexity, Morpho facilitates the querying, editing, and visualization of data collections. While it is no longer actively supported, Morpho remains accessible as an open-source application, providing continued availability for users (see https://knb.ecoinformatics.org/tools/morpho).

MDEmic (Kunis et al., 2021) presents a user-friendly platform for editing metadata associated with microscopic imaging data, offering a seamless experience. Simultaneously, it provides a high level of flexibility for adjusting metadata sets and their associated data models. In the context of the ongoing standardization process for metadata in microscopic experiments, MDEmic aligns itself with this evolving landscape, ensuring adaptability and compliance with emerging standards.

The SHAPEness Metadata Editor is a Java desktop application designed to assist users in creating and updating RDF metadata descriptions. Featuring a robust user interface, it facilitates the seamless population and validation of metadata structured as graphs (Paciello et al., 2022). This Metadata Editor has been developed within the framework of the European Plate Observing System (EPOS) (see https://epos-eu.github.io/SHAPEness-Metadata-Editor/gitpage/index.html).

ESPERANTO, developed in 2023, represents an innovative framework facilitating standardized semi-supervised harmonization and integration of toxicogenomics metadata, thereby enhancing their FAIRness in compliance with Good Laboratory Practice (Di Lieto et al., 2023). The tool ensures harmonization across metadata through the establishment of a specialized vocabulary. With a user-friendly interface, ESPERANTO is designed to support users in harmonizing metadata, irrespective of their background or expertise, providing a seamless experience.

SMetaS (Sample Metadata Standardizer, Bremer and Fiehn, 2023) is another novel software tool that is used for automated metadata standardization. Users construct a sample metadata matrix and populate it with natural language descriptions. Subsequently, the tool employs advanced algorithms to convert the matrix by substituting free-text terms with predefined vocabulary terms. This conversion process prioritizes simplicity and employs sophisticated techniques such as synonym matching and typographical correction within an n-grams/nearest neighbors model framework. SMetaS facilitates the downstream analysis of research studies and samples through the implementation of string equality, ensuring that data is FAIR for retrospective purposes.

Schema.org is a project focused on standardizing metadata vocabulary to enhance the FAIR principles of web content (Cano et al., 2022). Its application offers content creators the means to improve the accessibility and interoperability of their content. While leveraging schema.org can be advantageous for biomedical research resource providers, applying its standards to biomedical research outputs may present challenges. Nevertheless, Schema.org serves as a valuable tool for authoring, extending, and utilizing metadata schemas, ultimately contributing to the improvement of FAIRness in biomedical data.

NExtSEEK (Pradhan et al., 2022) empowers users to gather and organize essential information, enabling researchers to enhance reusability and reproducibility. It facilitates the dissemination of data and metadata to the scientific community through public repositories. NExtSEEK serves as a valuable tool to streamline the sharing and accessibility of research information.

The Clinical Data Interchange Standards Consortium's (CDISC) Operational Data Model (ODM) plays a crucial role as a flexible standard for the transmission and preservation of metadata and subject clinical data within the realm of clinical trials. However, due to the limited compatibility of some electronic health systems with ODM as an input format, there is a pressing need for the conversion of ODM to alternative data standards and formats. Addressing this challenge, ODMToolBox (Soto-Rey et al., 2018) offers a systematic template-driven approach for the development of ODM converters. By providing online access to templates, programming tools, and an ODM test suite, ODMToolBox simplifies the process of creating new converters, thereby promoting enhanced interoperability in the management of clinical trial data.

### 3.4 Data management tools

In our classification, instruments falling outside the three primary groups are categorized as Data Management Tools, all of which are integral to data stewardship. Within this classification, Data Management Tools can be further delineated into three subgroups: tools for data management planning, tools for measuring data FAIRness, and tools for managing data workflows.

**Data management planning tools** are essential instruments designed to assist researchers in developing strategies for organizing their data throughout the research lifecycle. It serves to create a data management plan (DMP) prior to the research.

The Data Stewardship Wizard (DSW) tool offers a user-friendly platform for customizing the DSW knowledge model, structured into chapters that encompass various facets of data management (Devignes et al., 2023). Within each chapter, specific sections house targeted questions designed to gather pertinent information. These questions are categorized based on their relevance to different stages of the data/project lifecycle and their impact on ensuring compliance with FAIR principles. Through an intuitive questionnaire interface, users can instantiate a knowledge model as a DMP project. Additionally, the tool allows for the preservation of pre-filled project versions as templates, facilitating the streamlined creation of multiple DMP projects that share common information.

Argos, developed by OpenAIRE, stands as a DMP tool integrated with other OpenAIRE services and the European Open Science Cloud (Papadopoulou et al., 2023). This tool facilitates the creation and editing of DMPs while promoting FAIR principles for sharing. It provides a workspace for convenient versioning of DMPs, accommodating changes that may occur throughout the research cycle.

The DMP Tool (Sallans and Donnelly, 2012), developed by the California Digital Library, is a free tool designed to assist researchers in creating Data Management Plans (DMPs). Offering guidance tailored to specific funders with DMP requirements, the tool is versatile and can be utilized by anyone seeking to develop generic DMPs for research facilitation. Additionally, the tool provides access to resources and services available at participating institutions to support the fulfillment of data management requirements.

EasyDMP (Philipson et al., 2023), developed by Sigma2 in collaboration with EUDAT2020, is a free-of-charge tool accessible to researchers in Norway and across Europe. The primary objective of EasyDMP is to offer researchers with limited experience in data management a straightforward method for creating a DMP. Achieving this goal involves translating the data management guidelines provided by funding agencies into a series of easy-to-answer questions, many of which include predefined responses. The resulting DMP serves as a blueprint for researchers to implement the necessary elements ensuring the proper management of their data.

**Tools for data FAIRness measurement** offer automated solutions for assessing and scoring the level of FAIRness using a given dataset. By employing various quantifiable metrics, these tools offer a comprehensive analysis of how well a dataset aligns with the FAIR principles. For instance, they may assess the presence of metadata that enhances findability, evaluate access protocols that ensure data can be easily retrieved, analyze the degree of interoperability with other datasets, and measure the reusability of data through licensing and documentation.

F-UJI (Devaraju and Huber, 2021) is specifically crafted for programmatically measuring the FAIR aspects of research data. Adhering to best practices, standards, and relevant literature in research data preservation and publication, the tool has undergone testing with pilot data repositories as part of the FAIRsFAIR project. Currently used by various projects within the European Open Science Cloud, F-UJI aims to contribute to the ongoing advancement of FAIR data principles in the research community.

The FAIRshake toolkit (Clarke et al., 2019) was created to facilitate the development of community-driven FAIR metrics, coupled with both manual and automated FAIR assessments. The outcomes of FAIR assessments are represented as insignias, which can be embedded within websites hosting digital resources. Utilizing FAIRshake, a range of biomedical digital resources underwent comprehensive evaluations, encompassing both manual and automated assessments, to gauge their degree of adherence to FAIR principles.

The FAIR Evaluation and Validation Assessment (EVA) tool, developed in the framework of the European Open Science Cloud, is tailored for data management systems such as open repositories. Its customizable nature enables seamless integration into diverse settings, offering scalability and automation. Designed to be adaptable across various environments, repository platforms, and scientific disciplines, FAIR EVA prioritizes the adherence to the dynamic FAIR Principles. Through FAIR EVA, data FAIRness can be quantitatively assessed, providing a valuable metric for evaluating data management practices (Aguilar Gómez and Bernal, 2023).

FAIR Evaluator (Wilkinson et al., 2019) is a system that uses measurable indicators, open-source tools, and community participation to evaluate digital resources. This system helps data stewards understand how FAIR their resources are and provides a roadmap for improvement.

FAIR-Checker (Gaignard et al., 2023) is an online tool that helps assess how FAIR a digital resource's metadata is. It has two main features: “Check” which evaluates metadata and gives suggestions for improvement, and “Inspect” which helps users directly enhance their metadata quality. Using Semantic Web technologies, FAIR-Checker automatically checks various FAIR metrics and lets users know what metadata is missing or needed to make their resource more FAIR.

FOOPS! (Garijo et al., 2021) is a web service that evaluates FAIRness of OWL ontology or SKOS [Simple Knowledge Organization System (Tomaszuk and Szeremeta, 2020)] thesaurus. It runs 24 checks across the different FAIR principles, looking at things like whether the ontology has a persistent identifier, uses open protocols, references other vocabularies, and provides clear documentation.

O'FAIRe is a framework for evaluating the FAIRness of ontologies (Amdouni et al., 2022). It utilizes 61 questions primarily focused on metadata descriptions, leveraging standard metadata properties to enhance the FAIRness of semantic resources.

**Data workflow tools** are software applications or platforms designed to streamline and automate the processes involved in managing, processing, and analyzing research data throughout its lifecycle.

MOLGENIS Research is an open-source web application designed to facilitate the collection, management, analysis, visualization, and sharing of large and intricate biomedical datasets. One of its notable features is its user-friendly interface, enabling users without advanced bioinformatics skills to work with complex data effectively. This tool caters to the diverse needs of biomedical researchers, providing a comprehensive platform for handling various aspects of data management and analysis in the field (van der Velde et al., 2019).

KNIME (see https://www.knime.com) provides a user-friendly environment that simplifies the creation of analytic models and task automation without the need for coding. The platform's no-code/low-code approach lowers the barriers to entry for data science, granting users access to advanced algorithms applicable to large datasets. KNIME's flexibility is enhanced by its support for various programming languages, enabling users to script custom algorithms through built-in integrations with languages such as R, Python, Java, and others. This versatility allows for customization to meet specific analytical needs. KNIME is widely used in life sciences (Fillbrunn et al., 2017). KNIME-CDK (Beisken et al., 2013) is a set of functionalities within KNIME that focuses on molecule-related operations, including conversion to and from common formats, generation of molecular signatures, fingerprints, and properties. Leveraging the capabilities of the Chemistry Development Toolkit (CDK), KNIME-CDK utilizes the Chemical Markup Language (CML) for persistence, providing a robust and versatile framework for handling chemical and molecular data within the KNIME analytics platform. This integration enhances KNIME's capabilities in cheminformatics and molecular data analysis.

iRODS (integrated rule-oriented data system) offers a rule-based system management approach, simplifying data replication and enhancing data protection. In contrast to the metadata provided by traditional file systems, iRODS features a comprehensive metadata system that enables users to customize application-level metadata according to their specific needs. This flexibility and rule-based management make iRODS a powerful solution for efficiently handling and protecting data, particularly in scenarios that require advanced data management and replication capabilities (Chiang et al., 2011).

In bioinformatics, Taverna workflows find common application in high-throughput omics analyses, such as proteomics or transcriptomics, as well as in evidence gathering methods involving text mining and data mining. Taverna provides scientists with access to a diverse set of tools and resources, numbering in the thousands, freely available from various life science institutions. Despite no longer being actively supported, Taverna continues to serve as a valuable tool in the field of life science informatics, offering a versatile platform for the design and execution of complex computational workflows (Wolstencroft et al., 2013).

The ISA (Investigation-Study-Assay) Software Suite (Rocca-Serra et al., 2010) is a collection of open-source software tools designed to facilitate the management, curation, and exchange of experimental metadata in life sciences research. The suite is particularly focused on providing solutions for representing metadata related to genomics and other high-throughput experiments. The ISA Software Suite is widely used in the life sciences community. Researchers and data curators often use ISA-Tab files to describe their experiments before submitting data to public repositories.

The ODAM Software Suite serves as an experimental platform for managing data tables, aiming to enhance the accessibility and reusability of research data with minimal input from data providers (Jacob et al., 2020). Tailored for user-friendly management of experimental data tables, ODAM offers a structured model for organizing both data and metadata, streamlining data handling and analysis processes. Additionally, ODAM aligns with FAIR principles, promoting data dissemination by fostering interoperability and reusability for both human users and automated systems. This framework enables comprehensive exploration and extraction of datasets, facilitating their utilization in entirety or selectively as per requirements (see https://inrae.github.io/ODAM/).

The Open Science Framework (Foster and Deardorff, 2017) functions as a facilitative tool, advocating for open and centralized workflows in the research lifecycle. It supports the comprehensive capture of various facets and outcomes of the research process, encompassing the development of research ideas, study design, storage and analysis of collected data, as well as the creation and publication of reports or papers.

OpenBIS stands as an open-source software framework designed for the development of user-friendly, scalable, and robust information systems tailored for handling data and metadata from biological experiments. It empowers users to gather, integrate, share, publish data, and establish connections to data processing pipelines. With the flexibility to be extended and customized, openBIS accommodates various data types acquired through diverse technologies (Bauch et al., 2011).

OpenRefine is a robust and freely available open-source tool crafted for managing unruly data. It serves multiple purposes, including cleaning and refining data, transforming it from one format to another, and augmenting it through web services and external data sources (Petrova-Antonova and Tancheva, 2020).

Researchers use Elsevier's Pure data repository to simplify and promote the data deposit process. Pure, as a Research Information Management System (Conte et al., 2017), is designed to be user-friendly and turnkey. Its deep integration into the Research Intelligence portfolio, along with external Open Access databases and Open Data repositories, facilitates actionable analysis across sources for improved decision-making and evidence-based execution of research strategy (see https://www.elsevier.com/products/pure).

SciCat (see https://scicatproject.github.io) is a scientific data management and annotation tool designed to implement a FAIR data management policy. As a fully open-source project, SciCat allows for easy extension of functionality through the RESTful OpenAPI.

Galaxy (Afgan et al., 2018) serves as a widely adopted web-based scientific analysis platform, catering to a global community of tens of thousands of scientists engaged in analyzing extensive biomedical datasets, including genomics, proteomics, metabolomics, and imaging. The platform is designed to tackle key challenges in data-driven biomedical science, emphasizing universal accessibility for researchers, ensuring the full reproducibility of analyses, and simplifying the communication of analyses to facilitate seamless reuse and extension.

## 4 Discussion

### 4.1 Future requirements for data stewardship tools

Very large quantities of data are being generated in scientific research and medicine, which presents challenges in terms of ensuring data accuracy and preventing the spread of false information. These challenges have implications for both scientific research and society at large. Therefore, it is essential to establish mechanisms to address these risks and safeguard against potential harm (Mons et al., 2017). It is crucial to improve the infrastructure and methods for “distributed learning” and ensure that the algorithms and services used are compatible with accurate and reliable metadata, and ideally, with FAIR data. The focus should be on the policy of distributing the data as widely as possible while centralizing it only when necessary (Mons et al., 2017). Also, while data continues to grow in volume, complexity, and diversity, one of the most important future requirements for data stewardship tools is scalability. Tools must be able to handle large and complex datasets. To fully use the potential of artificial intelligence in research and innovation, it is essential to ensure that data is made FAIR, which involves automating operations that support findability, accessibility, interoperability, and reusability (Schultes et al., 2022). There are tools that measure progress toward FAIR research data such as F-UJI (Devaraju and Huber, 2021) and FAIRshake (Clarke et al., 2019), but the demand for tools that can help ease the process of making data FAIR is quite high (Devaraju and Huber, 2021). Another requirement worth mentioning is the need to improve integration and interoperability. As data becomes more heterogeneous and the number of data sources grows, it can be challenging to integrate data from different sources or to compare data coming from different research (Watford et al., 2019). The lack of standards in the naming and measuring units is one of many problems of data integration. This requires the development of data integration tools and standards that can facilitate data interoperability.

## 5 Conclusion

The subject of data stewardship tools is fast developing, and there are a wide range of alternatives available to aid researchers in managing, sharing, and reusing research data. These instruments cover a broad variety of functionalities and are intended for use by researchers at different points in the research data lifecycle. Although there are more tools available than ever before, there are still open challenges that must be solved, including the need for standardization and interoperability among tools as well as for more efficient and automated workflows. Additionally, it is anticipated that new data stewardship tools will continue to appear as data volume and complexity increase, and that existing tools will need to be updated and improved to keep up with shifting data management requirements.
