# Supplementary material for: Current state of data stewardship tools in life science
Source: Front Big Data. 2024 Sep 16;7:1428568. doi: 10.3389/fdata.2024.1428568 (PMC11439729; doi:10.3389/fdata.2024.1428568)
Supplement: Supplementary file 1 [file Table_1.docx]

# Supplementary Material

| Tool | Project Link | Direct Link |
| --- | --- | --- |
| ADataViewer | [ADataViewer](https://adata.scai.fraunhofer.de) | [Study Picker](https://adata.scai.fraunhofer.de/study_picker) |
| Gene Expression Omnibus | [Gene Expression Omnibus](https://www.ncbi.nlm.nih.gov/geo/) | [Gene Expression Omnibus](https://www.ncbi.nlm.nih.gov/geo/) |
| Sequence Read Archive | [Sequence Read Archive](https://www.ncbi.nlm.nih.gov/sra) | [Sequence Read Archive](https://www.ncbi.nlm.nih.gov/sra) |
| European Genome-phenome Archive | [European Genome-phenome Archive](https://ega-archive.org) | [European Genome-phenome Archive](https://ega-archive.org) |
| GenBank | [GenBank](https://www.ncbi.nlm.nih.gov/genbank/) | [GenBank](https://www.ncbi.nlm.nih.gov/genbank/) |
| PRIDE | [PRIDE](https://www.ebi.ac.uk/pride/) | [PRIDE](https://www.ebi.ac.uk/pride/) |
| MassIVE | [MassIVE](https://massive.ucsd.edu/) | [Search](https://massive.ucsd.edu/ProteoSAFe/datasets.jsp#%7B%22query%22%3A%7B%7D%2C%22table_sort_history%22%3A%22createdMillis_dsc%22%7D) |
| PASSEL | [PASSEL](https://peptideatlas.org/passel/) | [Search](https://db.systemsbiology.net/sbeams/cgi/PeptideAtlas/GetSELTransitions) |
| Panorama Public | [Panorama Public](https://panoramaweb.org/home/project-begin.view?) | [Search](https://panoramaweb.org/Panorama%20Public/project-begin.view) |
| Image Data Resource | [Image Data Resource](https://idr.openmicroscopy.org) | [Image Data Resource](https://idr.openmicroscopy.org) |
| Cell Image Library | [Cell Image Library](http://www.cellimagelibrary.org/home) | [Search](http://www.cellimagelibrary.org/images/advanced_search) |
| EMPIAR | [EMPIAR](https://www.ebi.ac.uk/empiar/) | [Search](https://www.ebi.ac.uk/emdb/search/database:EMPIAR) |
| SSBD | [SSBD](https://ssbd.qbic.riken.jp) | [Search](https://ssbd.qbic.riken.jp/search3/) |
| BioImage Archive | [BioImage Archive](https://www.ebi.ac.uk/bioimage-archive/) | [Search](https://www.ebi.ac.uk/biostudies/bioimages/studies?query=) |
| Integrated Resource for Reproducibility in Macromolecular Crystallography | [Integrated Resource for Reproducibility in Macromolecular Crystallography](https://proteindiffraction.org) | [Search](https://proteindiffraction.org/browse/) |
| BioMagResBank | [BioMagResBank](https://bmrb.io) | [Search](https://bmrb.io/software/query) |
| EMDataBank | [EMDataBank](https://www.ebi.ac.uk/emdb/) | [Search](https://www.ebi.ac.uk/emdb/search/?q=) |
| Protein Data Bank | [Protein Data Bank](https://www.rcsb.org) | [Search](https://www.rcsb.org/search/advanced) |
| Zenodo | [Zenodo](https://zenodo.org) | [Search](https://zenodo.org/search?q=&l=list&p=1&s=10&sort=newest) |
| Open Science Framework | [Open Science Framework](https://osf.io) | [Search](https://osf.io/search?activeFilters=%5B%5D&q=&sort=-relevance&view_only=) |
| FAIRsharing | [FAIRsharing](https://fairsharing.org) | [FAIRsharing](https://fairsharing.org) |
| re3data | [re3data](https://www.re3data.org) | [re3data](https://www.re3data.org) |
| Dryad | [Dryad](https://datadryad.org/stash) | [Dryad](https://datadryad.org/stash) |
| FigShare | [FigShare](https://figshare.com) | [FigShare](https://figshare.com) |
| DataCite | [DataCite](https://datacite.org) | [DataCite Fabrica](https://doi.datacite.org) |
| Ontology Lookup Service | [Ontology Lookup Service](https://www.ebi.ac.uk/ols4) | [Ontologies](https://www.ebi.ac.uk/ols4/ontologies) |
| Nanopub Jupiter Lab Extension | [Github link](https://github.com/fair-workflows/NanopubJL) | [Github link](https://github.com/fair-workflows/NanopubJL) |
| ROHub | [ROHub](https://www.rohub.org) | [Create New RO](https://www.rohub.org/myros?activetab=overview) |
| OpenAIRE CONNECT | [OpenAIRE CONNECT](https://connect.openaire.eu) | [Get started](https://connect.openaire.eu/get-started) |
| SODAR | [SODAR](https://www.cubi.bihealth.org/software/sodar/) | [Github link](https://github.com/bihealth/sodar-server) |
| REDCap | [REDCap](https://www.project-redcap.org) | [Try REDCap](https://projectredcap.org/software/try/) |
| Aber-OWL | [Aber-OWL](http://aber-owl.net/#/) | [Aber-OWL](http://aber-owl.net/#/) |
| ukbREST | [PubMed Publication](https://pubmed.ncbi.nlm.nih.gov/30395166/) | [Github link](https://github.com/hakyimlab/ukbrest) |
| Figshare Harvester | [Github link](https://github.com/OpenVIVO/figshare-rdf) | [Github link](https://github.com/OpenVIVO/figshare-rdf) |
| FAIR data pipeline | [FAIR data pipeline](https://www.fairdatapipeline.org) | [Quick start](https://www.fairdatapipeline.org/docs/quick_start/) |
| Amnesia | [Amnesia](https://amnesia.openaire.eu) | [Get Amnesia](https://amnesia.openaire.eu/download.html) |
| µ-ANT | [PubMed Publication](https://pubmed.ncbi.nlm.nih.gov/31621826/) | [Github link](https://github.com/CrisesUrv/microaggregation-based_anonymization_tool) |
| Anonimatron | [Anonimatron](https://realrolfje.github.io/anonimatron/) | [Github link](https://github.com/realrolfje/anonimatron) |
| Anonymizer | [Github link](https://github.com/DivanteLtd/anonymizer) | [Github link](https://github.com/DivanteLtd/anonymizer) |
| NLM-Scrubber | [NLM-Scrubber](https://lhncbc.nlm.nih.gov/scrubber/) | [Download](https://lhncbc.nlm.nih.gov/scrubber/download.html) |
| Anonymeter | [Anonymeter](https://www.anonos.com/products/anonymeter) | [Github link](https://github.com/statice/anonymeter) |
| AMBIENT | [PubMed Publication](https://pubmed.ncbi.nlm.nih.gov/34450935/) | [PubMed Publication](https://pubmed.ncbi.nlm.nih.gov/34450935/) |
| GDPRValidator | [PubMed Publication](https://pubmed.ncbi.nlm.nih.gov/36532816/) | [Zenodo link](https://zenodo.org/records/7224749) |
| Automated GDPR Compliance Verification Tool | [PubMed Publication](https://pubmed.ncbi.nlm.nih.gov/35408377/) | [PubMed publication](https://pubmed.ncbi.nlm.nih.gov/35408377/) |
| GDPR Toolkit for Digital Health | [PubMed Publication](https://pubmed.ncbi.nlm.nih.gov/35673005/) | [PubMed Publication](https://pubmed.ncbi.nlm.nih.gov/35673005/) |
| OxO | [OxO](https://www.ebi.ac.uk/spot/oxo/) | [OxO](https://www.ebi.ac.uk/spot/oxo/) |
| UMLS MetaMap | [UMLS MetaMap](https://lhncbc.nlm.nih.gov/ii/tools/MetaMap.html?_gl=1*1smc6l6*_ga*NTE2NjQ4ODUyLjE3MTgxMzQxMTU.*_ga_7147EPK006*MTcyMTk5Njg3MC4xLjAuMTcyMTk5Njg3MC4wLjAuMA..*_ga_P1FPTH9PL4*MTcyMTk5Njg3MC4xLjAuMTcyMTk5Njg3MC4wLjAuMA..) | [Use MetaMap](https://lhncbc.nlm.nih.gov/ii/tools/MetaMap/use-MetaMap.html) |
| BioPortal | [BioPortal](https://bioportal.bioontology.org) | [Search](https://bioportal.bioontology.org/search) |
| BiobankConnect | [PubMed Publication](https://pubmed.ncbi.nlm.nih.gov/25361575/) | [Repository link](https://central.sonatype.com/artifact/org.molgenis/molgenis-omx-biobankconnect) |
| Karma | [Karma](https://usc-isi-i2.github.io/karma/) | [Github link](https://github.com/usc-isi-i2/Web-Karma) |
| RightField | [RightField](https://rightfield.org.uk) | [Download](https://rightfield.org.uk/download) |
| SCALEUS FD | [PubMed Publication](https://pubmed.ncbi.nlm.nih.gov/32908882/) | [Github link](https://github.com/bioinformatics-ua/scaleus-fair) |
| Tyto | [PubMed Publication](https://pubmed.ncbi.nlm.nih.gov/35226470/) | [Github link](https://github.com/SynBioDex/tyto) |
| Data Steward Tool | [Data Steward Tool](https://data-steward.bio.scai.fraunhofer.de/data-steward) | [Github link](https://github.com/SCAI-BIO/data-steward) |
| ISA API | [ISA API](https://isa-tools.org/isa-api/content/index.html) | [Github link](https://github.com/ISA-tools/isa-api) |
| FAIR data station | [FAIR data station](https://fairds.fairbydesign.nl) | [Metadata configurator](https://fairds.fairbydesign.nl/configurator) |
| CEDAR | [CEDAR](https://more.metadatacenter.org) | [CEDAR](https://more.metadatacenter.org) |
| Qvain | [Qvain](https://qvain.fairdata.fi) | [Describe dataset](https://qvain.fairdata.fi/dataset) |
| Morpho | [Morpho](https://knb.ecoinformatics.org/tools/morpho) | [Github link](https://github.com/NCEAS/morpho) |
| MDEmic | [PubMed Publication](https://www.ncbi.nlm.nih.gov/pmc/articles/PMC9514507/) | [Github link](https://github.com/ome/omero-insight) |
| SHAPEness Metadata Editor | [SHAPEness Metadata Editor](https://epos-eu.github.io/SHAPEness-Metadata-Editor/gitpage/index.html) | [Download](https://github.com/epos-eu/SHAPEness-Metadata-Editor/releases) |
| ESPERANTO | [Bioinformatics Publication](https://academic.oup.com/bioinformatics/article/39/6/btad405/7206881) | [Github link](https://github.com/fhaive/esperanto) |
| SMetaS | [PubMed Publication](https://pubmed.ncbi.nlm.nih.gov/37623884/) | [Github link](https://github.com/metabolomics-us/metadatastandardizer) |
| Schema.org | [schema.org](https://schema.org) | [schema.org](https://schema.org) |
| NExtSEEK | [PubMed Publication](https://pubmed.ncbi.nlm.nih.gov/35836998/) | [Github link](https://github.com/BMCBCC/NExtSEEK) |
| ODMToolBox | [ODMToolBox](https://www.medizin.uni-muenster.de/en/imi/forschung/digital-health/odmtoolbox.html) | [ODMToolBox](https://www.medizin.uni-muenster.de/en/imi/forschung/digital-health/odmtoolbox.html) |
| Data Stewardship Wizard | [Data Stewardship Wizard](https://ds-wizard.org) | [Get started](https://ds-wizard.org/get-started) |
| Argos | [Argos](https://argos.openaire.eu/splash/) | [Start your DMP](https://argos.openaire.eu/home) |
| DMPTool | [DMPTool](https://dmptool.org) | [Get started](https://dmptool.org/auth) |
| EasyDMP | [EasyDMP](https://easydmp.no) | [EasyDMP](https://easydmp.no) |
| F-UJI | [F-UJI](https://www.f-uji.net) | [Assess a dataset](https://www.f-uji.net/?action=test) |
| FAIRshake | [FAIRshake](https://fairshake.cloud) | [FAIRshake](https://fairshake.cloud) |
| FAIR EVA | [FAIR EVA](https://www.faireva.org) | [Github link](https://github.com/EOSC-synergy/FAIR_eva) |
| FAIR Evaluator | [FAIR Evaluator](https://fairsharing.github.io/FAIR-Evaluator-FrontEnd/#!/) | [Evaluations](https://fairsharing.github.io/FAIR-Evaluator-FrontEnd/#!/evaluations) |
| FAIR-Checker | [FAIR-checker](https://fair-checker.france-bioinformatique.fr) | [Check](https://fair-checker.france-bioinformatique.fr/check) |
| FOOPS! | [FOOPS!](https://foops.linkeddata.es/FAIR_validator.html) | [FOOPS!](https://foops.linkeddata.es/FAIR_validator.html) |
| O'FAIRe | [Publication link](https://hal.science/lirmm-03630543/) | [Github link](https://github.com/agroportal/fairness) |
| MOLGENIS | [MOLGENIS](https://molgenis.org) | [Releases](https://github.com/molgenis/molgenis-emx2/releases) |
| KNIME | [KNIME](https://www.knime.com) | [Download](https://www.knime.com/downloads/download-knime?token=1722001304:46.114.88.34:4267e0ffa5bf61748c767f01a0a4c8b804c65d33c536ea3a3aa3e7051f0aaf9c) |
| iRODS | [iRODS](https://irods.org) | [Download](https://irods.org/download/) |
| Taverna | [Taverna](https://incubator.apache.org/projects/taverna.html) | [Download](http://www.taverna.org.uk/download/workbench/) |
| ODAM | [ODAM](https://inrae.github.io/ODAM/) | [Installation](https://inrae.github.io/ODAM/install/) |
| Open Science Framework | [OSF](https://osf.io) | [Get started](https://osf.io/register?campaign=&next=&view_only=) |
| OpenBIS | [OpenBIS](https://openbis.ch/) | [Download](https://openbis.ch/index.php/downloads/#downloads) |
| OpenRefine | [OpenRefine](https://openrefine.org) | [Download](https://openrefine.org/download) |
| Pure | [Pure](https://www.elsevier.com/products/pure) | [Pure](https://www.elsevier.com/products/pure) |
| SciCat | [SciCat](https://scicatproject.github.io) | [Github link](https://github.com/ScicatProject) |
